# A Brief Educational Pre-exposure Prophylaxis Intervention in an Infectious Disease Clinic: Protocol for a Case Series Study

**DOI:** 10.2196/33093

**Published:** 2022-11-23

**Authors:** Cynthia Dalton, Judith Cornelius, Bernard Davis

**Affiliations:** 1 University of North Carolina at Charlotte Charlotte, NC United States; 2 RAO Community Health Charlotte, NC United States

**Keywords:** PrEP, men who have sex with men, protocol, case series design, HIV, pre-exposure prophylaxis, sexual health, HIV prevention, health education, educational intervention

## Abstract

**Background:**

Black men who have sex with men (BMSM) remain the highest group infected with HIV despite treatment with medications known as pre-exposure prophylaxis (PrEP). PrEP in combination with safer sex practices has shown efficacy in preventing HIV infection. Despite awareness campaigns, PrEP uptake remains low among BMSM. While brief educational interventions have value in fast-paced clinical settings with limited appointment times, a brief PrEP educational intervention has not been initiated with BMSM in a fast-paced outpatient infectious disease clinic in North Carolina.

**Objective:**

The purpose of this study was to examine the effect of initiating a brief PrEP educational intervention to reduce HIV infection rates in BMSM in a fast-paced infectious disease clinic delivered by a doctoral-prepared nurse practitioner.

**Methods:**

This case-series study uses a brief educational intervention to develop and pilot-test a brief PrEP educational uptake intervention with BMSM. The participants met with the nurse practitioner at 3 different time points: baseline, 4 weeks later (first visit), and at the 3-month follow-up (second visit). We used a pretest-posttest design to examine the primary outcomes of PrEP knowledge, medication adherence, and sexually transmitted infection outcomes.

**Results:**

Due to the COVID-19 pandemic, the recruitment process was delayed. From November 1, 2019, to August 30, 2021, a total of 7 participants consented to participate in the study. Data analysis will be completed by the end of September 2022. We will submit a manuscript for publication consideration by December 2022.

**Conclusions:**

Brief educational interventions delivered in a fast-paced infectious disease clinic have the potential to increase PrEP awareness and knowledge, medication adherence, and decreased rates of sexually transmitted diseases in BMSM. This protocol will contribute to the literature on the development of brief PrEP educational interventions and has the potential to be generalized to other populations (eg, women and adolescents).

**International Registered Report Identifier (IRRID):**

RR1-10.2196/33093

## Introduction

### Background

Despite advances in HIV prevention care, Black men who have sex with men (BMSM) living in the southern region of the United States remain at a much higher risk of acquiring HIV when compared to other racial or ethnic groups [1,2]. BMSM living in this region of the country have been historically marginalized, have greater unmet social determinants of health needs, and have higher rates of HIV infection [3]. One of the southern states targeted for HIV prevention efforts is North Carolina [1]. In 2019, North Carolina was the sixth-highest state for new HIV diagnoses, with higher rates among racial and ethnic groups [4].

Widespread lack of awareness and knowledge of pre-exposure prophylaxis (PrEP) among BMSM in the United States persists. Despite significant evidence of efficacy, there are barriers to PrEP awareness and uptake. First, there is a limited understanding of PrEP among health care professionals [5]. Few studies demonstrate that PrEP works as long as it is taken as prescribed, making adherence a challenge [6]. The successful implementation of PrEP is driven by four elements: (1) linkage of BMSM to PrEP providers, (2) access to PrEP medications, (3) adherence to the prescribed regimen, and (4) ongoing sexual risk reduction behaviors [7]. Another factor to consider with PrEP uptake is the history of medical mistrust in the African American community (eg, the Tuskegee experiment) [8]. One way to link BMSM to the PrEP care continuum is through the acceleration of new models of care with nurse practitioners (NPs).

### Project Goal

The long-term goal of this study is to increase the rate of PrEP uptake in HIV-negative BMSM. The primary objective of this study was to develop a study protocol for a brief educational PrEP intervention delivered by a doctoral-prepared NP in a fast-paced infectious disease clinic. The success of PrEP uptake is dependent on behavioral variables such as knowledge of PrEP; willingness to take PrEP; and acceptability of, readiness for, and adherence to PrEP [7]. Understanding the demographic and behavioral predictors of intentions to use PrEP proved useful in identifying prospective participants for this study [7,8].

## Methods

### Design

For this pilot project, a case-series design was used to determine the impact of the integration of a PrEP protocol in HIV-negative BMSM. A case series is a group or series of case reports involving patients who are given similar treatment [9,10]. Case study data can include demographic information such as age, gender, ethnic origin, as well as information on diagnosis, treatment, response to treatment, and follow-up after treatment [9,10].

### Sample

The target population was a convenience sample of HIV-negative BMSM who had engaged in anal sex without condoms or sex with men who have sex with men (MSM) diagnosed with a sexually transmitted infection (STI) in the past 6 months who received care from the infectious disease clinic. Inclusion criteria included the following: (1) participants had to be older than 18 years of age, (2) able to give consent, and (3) are not infected with hepatitis B or C. Exclusion criteria included being unable to provide consent, less than 18 years of age, HIV positive, and infected with hepatitis B or C. Due to the COVID-19 pandemic, the recruitment process was delayed. From November 2019 to August 2021, other health care professionals (nurses and pharmacists; n=4) informed prospective participants (HIV-negative MSM) about the study using the institutional review board (IRB)–approved recruitment flyer. Interested participants were referred to an infectious disease physician who screened for eligibility. Those eligible to participate in the study were scheduled and directed to the NP.

### Setting

The protocol was delivered in an infectious disease clinic at a large medical center in southern United States. This clinic has a large clientele of BMSM. Approximately 20 BMSM are diagnosed with HIV each month, noting the urgent need for prevention efforts. The clinic’s staff provides interprofessional services to more than 2000 patients seeking treatment for HIV prevention or treatment annually.

### The Brief Educational PrEP Intervention Protocol

The evaluation of candidacy for HIV PrEP [11] was used to guide the design and development of the PrEP protocol. Additionally, guidelines from the Centers for Disease Control and Prevention, a review of the literature, and input from the clinical staff (infectious disease doctors, NPs, nurses, social workers, and patient navigators) were included [1-7,11]. On the first scheduled visit, the NP discussed the PrEP protocol and data collection procedures (eg, consent, laboratory results, and surveys). After completing the 24-item attitudes and behavior toward PrEP among high-risk HIV seronegative MSM survey [12] and the pretest 9-item PrEP knowledge survey [6], the participants received a brief face-to-face educational intervention accompanied by a PrEP 101 handout given at the end of the session (see Table 1 and Multimedia Appendix 1). The educational intervention provided information on PrEP indications, side effects, and how to take the medication. At this visit, the baseline specimens were also collected. PrEP laboratory tests included HIV antigen–antibody testing, a comprehensive metabolic panel, a hepatitis panel, as well as syphilis, and, if required, gonorrhea and chlamydia testing at anatomical sites of exposure. A medication pill log was provided to each patient to allow them to record when they had taken their PrEP medication. Condoms were available for distribution at each clinic visit. Two days later, a prescription was sent to the participant’s pharmacy of choice after their laboratory results had been reviewed.

Before the follow-up sessions (4 weeks and 3 months from baseline), repeat laboratory results were ordered. At the follow-up session, the NP reviewed the laboratory results and the PrEP 101 handbook with each participant. Strategies to maintain PrEP adherence and identify the negative consequences of unprotected sexual encounters were emphasized, and condoms were distributed as needed. At the third visit, the 9-item PrEP knowledge survey was administered to assess PrEP knowledge retention (Table 1). The NP contacted the pharmacy to monitor prescription refills with each follow-up visit. The participants were given a US $25 gift card on the first visit and a US $25 gift card on the third visit. No monetary compensation was provided for the second visit (Table 1). Table 1 summarizes the measures used in this study.

**Table 1:** Description of protocol measures, expected outcomes, and assessment times.

Measure	Description	Outcome	Data collection
Evaluation of PrEP^a^ criteria screening tool	25-item survey to evaluate eligibility for PrEP	Screening tool for eligibility	Enrollment
Attitudes and behaviors toward PrEP among high-risk HIV-seronegative men who have sex with men	24 item: 5-point Likert scale, true or false, yes or no	Readiness to take PrEP	Enrollment
Demographics	6 questions: age, gender, education, race or ethnicity, and exposures	Sample characteristics	Baseline
Pre-/post-PrEP knowledge	9 questions: yes or no, true or false, select all what medications are used for PrEP, and what else should be used with PrEP to prevent HIV transmission	PrEP knowledge	Baseline Follow-up 1: 4 weeks Follow-up 2: 3 months
Medication log and pharmacy outreach	7-day weekly log sheet of when medication is taken	Medication adherence	Follow-up 1: 4 weeks Follow-up two: 3 months
Blood work and swabs	Rapid plasma reagin Gonorrhea and chlamydia test at anatomical sites of exposure	Sexually transmitted infections	Baseline Follow-up 1: 4 weeks Follow-up 2: 3 months
Blood work	Complete metabolic panel, hepatitis panel HIV antigen-antibody testing	Check kidney function, liver function, and HIV status	Baseline Follow-up 1:4 weeks Follow-up 2: 3 months

^a^PrEP: pre-exposure prophylaxis.

### Outcome Measures

We examined medication adherence, PrEP knowledge, and incidence of STIs that occurred while the participants were enrolled in the study. The number of participants on PrEP, those who stayed on PrEP, those who stopped taking PrEP, and those who tested positive for HIV while taking PrEP were examined. PrEP knowledge was measured with the 9-item PrEP knowledge survey (pretest and posttest), and medication adherence was measured by the number of participants who had their medications refilled and the number of dosages recorded taken on the medication log. STI data were obtained from the laboratory work (HIV testing and rapid plasma reagin blood specimen) and swabs (oral and rectal). Condom use was measured with the question: Over the past 3 months, did you use a condom with each sexual encounter? If they answered “no,” then the next question was “Over the past 3 months, how many times did you use a condom while having sex?” We encouraged the participants to be open and honest with their responses (Table 1).

### Data Collection, Management, and Analysis

All data were collected and managed using Excel (Microsoft Corporation) spreadsheets. The NP was the sole data collector for this study. As a result of the small sample size, all study outcomes were analyzed using descriptive statistics and inferential tests (*t* test, chi-square) to examine trends over time.

### Confidentiality

Participants were informed that safeguards were in place to protect confidentiality and anonymity. All study-related information and spreadsheets were stored in a locked office and file cabinet at the study site. All participant information was coded by ID number to maintain confidentiality. We protected confidentiality by removing identifiers as soon as possible. Only members of the research team have access to the data, and only aggregate data will be presented for publication [13].

### Ethics Approval

Approval from the Wake Forest University Institutional Review Board (IRB00052082) was required since the case study was categorized as a research study [9,10]. The study’s protocol, surveys, and informed consent forms were reviewed to ensure respect, fairness, and safety in human subjects research [13]. The protocol was followed in accordance with the standards for human subjects research. The study participants were given the opportunity to opt out and were informed of their right to privacy. Each member of the research team completed the required training on proper methods of conducting research in compliance with federal and state requirements [13].

### Harm

Because the participants were involved in a drug-related study, they were monitored for adverse effects. We defined an adverse event as an event that occurred during the study that resulted in physical, psychological, or social harm to the participant [13]. Upon giving consent, if a participant experienced an adverse event but did not start to receive PrEP, the event would be reported as not related to PrEP. If PrEP was discontinued as a result of an adverse event, the research team would record the event and data, leading to the discontinuation of the medication, which would be reported to the IRB. Adverse events that are life-threatening or extreme or require hospitalization will be reported to the IRB within 1 week of the event [13]. If a serious adverse event occurred after the study was discontinued, it will not be reported as an adverse effect unless the research team recognized that the event may have been caused by PrEP or the study protocol.

## Results

A total of 7 African American men consented to participate in the pilot study. Data analysis is to be completed by late September 2022. We will submit a manuscript for publication consideration by December 2022.

## Discussion

### Anticipated Findings

We hypothesize that the brief educational intervention will show an increase in medication adherence and PrEP knowledge and a decrease in the rates of STIs. The educational protocol for this study implored a multimodal approach. The combination of intervention approaches (1-on-1 education, handouts, etc) with a clinical outcome (HIV-negative status with PrEP uptake) has been shown to have the highest improvement in medication adherence [14].

Interventions with brief follow-up periods have been effective for long-term chronic medication adherence [12,15]. Similar to Centers for Disease Control and Prevention guidelines, the follow-up periods were 4 weeks and 3 months [12]. For a PrEP program to be effective, it must be accessible to those who would benefit the most from it [16]. In this study, the NP called the pharmacy to verify medication refills. An alternative to the calls to the pharmacy could be a web-based management system, which is effective for optimizing PrEP uptake with automatic refill SMS text messaging [17].

### Strengths and Limitations

One strength of this project is the setting. Currently, the state of North Carolina ranks in the top 10 states with high rates of STIs [4]. STIs have been known to be precursors to HIV infections. Consistent with the literature, another strength was the use of an infectious disease clinic with educated health care professionals to increase PrEP accessibility to those at high risk of HIV [12]. This setting offered treatment not only for HIV prevention but for STI treatment. Lastly, NPs are readily available to be included in new models of the PrEP care continuum [18].

There were 4 limitations to this study. The first limitation was the use of a convenience sample of HIV-negative men from 1 infectious disease clinic in a single geographic location; therefore, the findings cannot be generalized to other groups of HIV-negative men. The second limitation was the use of medication logs. The self-reporting of PrEP uptake does not ensure medication adherence [14]. While the NP confirmed that the medications were being refilled and picked up at the pharmacy, daily doses could be missed. The third limitation was that some patients may no longer see the need to take a daily dose of PrEP and could benefit from PrEP on demand if they were no longer in a committed relationship, which was not explored in this study. The fourth limitation was that the case study design is time-consuming and the findings cannot be generalized to a wider population. However, this design allows for greater depth in the data that other designs do not allow [19]. Nevertheless, this study provides insights into the use of a brief educational PrEP intervention in a fast-paced clinic.

### Future Directions

We developed a strategic dissemination plan in partnership with other infectious disease clinics in the health care setting. The research process and our findings will be shared with clinical staff, in a peer-reviewed journal, and at a research conference.

### Conclusions

This study will close the gap in identifying opportunities to deliver current HIV prevention education to minority MSM in a fast-paced clinical setting. The study’s findings will add to the current literature on the effect of a brief PrEP educational intervention on increasing PrEP knowledge, improving medication adherence, and reducing HIV seroconversion among BMSM. This study aligns with the End the Epidemic Plan for America to reduce the rates of HIV infections by 90% by the year 2030.

## Appendix

Multimedia Appendix 1 Pre-exposure Prophylaxis (PrEP) protocol handout.

## Abbreviations

BMSM: Black men who have sex with men
IRB: institutional review board
MSM: men who have sex with men
NP: nurse practitioner
PrEP: pre-exposure prophylaxis
STI: sexually transmitted infection
