# Supplementary material for: A Brief Educational Pre-exposure Prophylaxis Intervention in an Infectious Disease Clinic: Protocol for a Case Series Study
Source: JMIR Res Protoc. 2022 Nov 23;11(11):e33093. doi: 10.2196/33093 (PMC9730200; doi:10.2196/33093)
Supplement: Multimedia Appendix 1 [file resprot_v11i11e33093_app1.docx]

##### Multimedia Appendix 1. PrEP protocol handout.

##### PrEP 101

**Are** **you** **HIV-negative** **but** **at** **very** **high** **risk** **for** **HIV?** **Taking**  **PrEP** **every day** **can** **help** **keep** **you** **free** **from** **HIV.**

**What** **Is** **PrEP?**

- PrEP, or pre-exposure prophylaxis, is daily medicine that can reduce your chance of getting HIV.
- PrEP can stop HIV from taking hold and spreading throughout your body.
- Daily PrEP reduces the risk of getting HIV from sex by more than 90%. Among people who inject drugs, it reduces the risk by more than 70%.
- Your risk of getting HIV from sex can be even lower if you combine PrEP with condoms and other prevention methods.

##### Is PrEP Right For You?

- PrEP may benefit you if you are HIV-negative and **ANY** of the following apply to you.

##### You are a gay/bisexual man and

- have an HIV-positive partner.
- have multiple partners, a partner with multiple partners, or a partner whose HIV status is unknown–and you also
- have anal sex without a condom, or
- recently had a sexually transmitted disease (STD).

##### You are a heterosexual and

- have an HIV-positive partner.
- have multiple partners, a partner with multiple partners, or a partner whose HIV status is unknown–and you also
- don’t always use a condom for sex with people who inject drugs, or don’t always use a condom for sex with bisexual men.

##### You inject drugs and

##### share needles or equipment to inject drugs.

##### recently went to a drug treatment program. are at risk for getting HIV from sex.

##### Visit Your Healthcare Provider

##### To find out if PrEP is right for you.

##### Every 3 months, if you take PrEP, for repeat HIV tests, prescription refills, and follow-up

**If you don’t have a provider, visithttps://preplocator.org to locate one.**

**How** **Can** **You** **Get** **Help** **To** **Pay** **For** **PrEP?**

- Most private and state Medicaid plans cover PrEP. If you are on Medicaid, check with your benefits counselor.
- If you have health insurance, you may receive co-pay assistance from drug manufacturers or patient advocacy foundations.
- If you are without medical insurance, consider enrolling in an insurance marketplace, manufacturer patient assistance program, or your state’s Medicaid plan, if you are eligible for it.
- Learn more about paying for PrEP at [www.cdc.gov/hiv/pdf/risk/prep/cdc-hiv-](http://www.cdc.gov/hiv/pdf/risk/prep/cdc-hiv-) paying-for-prep.pdf.

**For** **more** **information** **please** **visit** [**www.cdc.gov/hiv**National](http://www.cdc.gov/hivNational) Center for HIV/AIDS, Viral Hepatitis, STD, and TB PreventionDivision of HIV/AIDS Prevention
